# Identification of a novel class of RIP1/RIP3 dual inhibitors that impede cell death and inflammation in mouse abdominal aortic aneurysm models

**DOI:** 10.1038/s41419-019-1468-6

**Published:** 2019-03-06

**Authors:** Ting Zhou, Qiwei Wang, Noel Phan, Jun Ren, Huan Yang, Conner C. Feldman, John B. Feltenberger, Zhengqing Ye, Scott A. Wildman, Weiping Tang, Bo Liu

**Affiliations:** 1 Department of Surgery, School of Medicine and Public Health, University of Wisconsin, Madison, WI 53705 USA; 2 School of Pharmacy, Medicinal Chemistry Center, University of Wisconsin, Madison, WI 53705 USA; 3 UW Carbone Cancer Center, School of Medicine and Public Health, University of Wisconsin, Madison, WI 53705 USA; 4 Department of Cellular and Regenerative Biology, School of Medicine and Public Health, University of Wisconsin, Madison, WI 53705 USA; 5 Present Address: Department of Cancer Biology, Dana-Farber Cancer Institute, Harvard Medical School, Boston, MA 02215 USA; 6 Present Address: Department of Radiation Oncology, Massachusetts General Hospital, Harvard Medical School, Boston, MA 02114 USA

## Abstract

Receptor interacting protein kinase-1 and -3 (RIP1 and RIP3) are essential mediators of cell death processes and participate in inflammatory responses. Our group recently demonstrated that gene deletion of *Rip3* or pharmacological inhibition of RIP1 attenuated pathogenesis of abdominal aortic aneurysm (AAA), a life-threatening degenerative vascular disease characterized by depletion of smooth muscle cells (SMCs), inflammation, negative extracellular matrix remodeling, and progressive expansion of aorta. The goal of this study was to develop drug candidates for AAA and other disease conditions involving cell death and inflammation. We screened 1141 kinase inhibitors for their ability to block necroptosis using the RIP1 inhibitor Necrostatin-1s (Nec-1s) as a selection baseline. Positive compounds were further screened for cytotoxicity and virtual binding to RIP3. A cluster of top hits, represented by GSK2593074A (GSK’074), displayed structural similarity to the established RIP3 inhibitor GSK’843. In multiple cell types including mouse SMCs, fibroblasts (L929), bone marrow derived macrophages (BMDM), and human colon epithelial cells (HT29), GSK’074 inhibited necroptosis with an IC50 of ~3 nM. Furthermore, GSK’074, but not Nec-1s, blocked cytokine production by SMCs. Biochemical analyses identified both RIP1 and RIP3 as the biological targets of GSK’074. Unlike GSK’843 which causes profound apoptosis at high doses (>3 µM), GSK’074 showed no detectable cytotoxicity even at 20 µM. Daily intraperitoneal injection of GSK’074 at 0.93 mg/kg significantly attenuated aortic expansion in two mouse models of AAA (calcium phosphate: DMSO 66.06 ± 9.17% vs GSK’074 27.36 ± 8.25%, *P* < 0.05; Angiotensin II: DMSO 85.39 ± 15.76% vs GSK’074 36.28 ± 5.76%, *P* < 0.05). Histologically, GSK’074 treatment diminished cell death and macrophage infiltration in aneurysm-prone aortae. Together, our data suggest that GSK’074 represents a new class of necroptosis inhibitors with dual targeting ability to both RIP1 and RIP3. The high potency and minimum cytotoxicity make GSK’074 a desirable drug candidate of pharmacological therapies to attenuate AAA progression and other necroptosis related diseases.

## Introduction

Receptor interacting protein kinase-1 and -3 (RIP1 and RIP3) are threonine/serine protein kinases that share a conserved kinase domain. The protein–protein interaction between RIP1 and RIP3 is an essential signaling step to initiate necroptosis in most cell types^1,2^. Necroptosis or programmed necrosis is increasingly recognized as a major cell death mechanism in conditions when the apoptotic pathway is compromised or in pathological sterile inflammation^3–5^. Additional to its role in necroptosis, RIP1, through its death domain, binds to death receptors including tumor-necrosis factor receptor 1 (TNF-R1) and triggers the formation of various signaling complexes that promote cell survival or apoptosis depending on the cell type and cellular contents^6,7^. In various cell types, RIP1 and RIP3 regulate expression of proinflammatory cytokines through mechanisms independent of necrosis and the subsequent release of danger-associated molecular pattern molecules (DAMPs)^8^. Targeted gene deletion of *Rip1* or *Rip3* has different developmental consequences. While *Rip1*^−/−^ mice die shortly after birth^9^, *Rip3*^−/−^ mice are vital^10^ and have proven to be instrumental in uncovering RIP3’s roles in vivo^11^.

Levels of RIP3 as well as RIP1 are elevated in human tissues affected by various pathological conditions including ischemic stroke, atherosclerosis, and aortic aneurysm^12–15^. In preclinical mouse models, gene deletion of *Rip3* as well as pharmacological inhibition of RIP1 alleviate disease severity^12,13,16,17^. Our lab demonstrates that in abdominal aortic aneurysm (AAA), RIP3 deficiency inhibits aneurysm formation via suppressing cell necrosis and inflammatory response of aortic smooth muscle cells (SMCs)^14^. Despite the perinatal lethality of RIP1 deficient mice, RIP1 kinase-dead knockin mice (K45A and D138N) are viable, and ameliorate cell death in intracerebral hemorrhage^18^ and TNF-induced shock model^19,20^. *Rip1* D138N mutant mice also showed beneficial effect in kidney ischemia—reperfusion injury, systemic inflammation associated with A20 deficiency^11^. How RIP1 kinase-dead mutations may affect cardiovascular diseases including atherosclerosis, stroke, and AAA has not yet been reported.

Since 2005, the discovery of Necrostatin-1 (Nec-1)^12^, the first proven RIP1 inhibitor^21^, appreciable efforts have been devoted to identification of small molecules with antagonizing activities against necroptosis^22^. Nec-1, as well as its improved version Nec-1s, is widely used to probe RIP1 functions in pathogenesis of multiple human disease models^22–24^. Administration of Nec-1 or Nec-1s in mice with brain ischemic injury^12^ or existing AAA^16^ proved in principle that blocking necroptosis may slow and even reverse disease progression. Small chemical inhibitors of necroptosis are also valuable tools to study RIP1 and RIP3 that have both kinase-dependent and -independent functions. Using a group of RIP3 inhibitors including GSK’843 and GSK’872, Mandal et al. uncovered a kinase-independent pro-apoptotic function of RIP3^25^. Although GSK’840, GSK’843, and GSK’872 are highly selective to RIP3, their in vivo use is limited due to their unique ability to promote assembly of a pro-apoptotic complex containing RIP3, RIP1, and caspase 8-FADD-cFLIP^25^.

In this study, we screened 3 libraries of kinase inhibitors with an intention to identify necroptosis inhibitors using a strategy that selects for potency, toxicity, and specificity. We identified a novel class of inhibitors represented by GSK2593074A (GSK’074), which completely blocked necroptosis in both human and murine cells at 10 nM. Biochemical and molecular docking analyses demonstrated that GSK’074 bound to and inhibited both RIP1 and RIP3 as a type II kinase inhibitor. Furthermore, this new inhibitor was well tolerated by mice and attenuated vascular inflammation and aortic expansion in two distinct AAA models and in both male and female mice.

## Results

### Discovery of a new class of necroptosis inhibitors

**Fig. 1:**
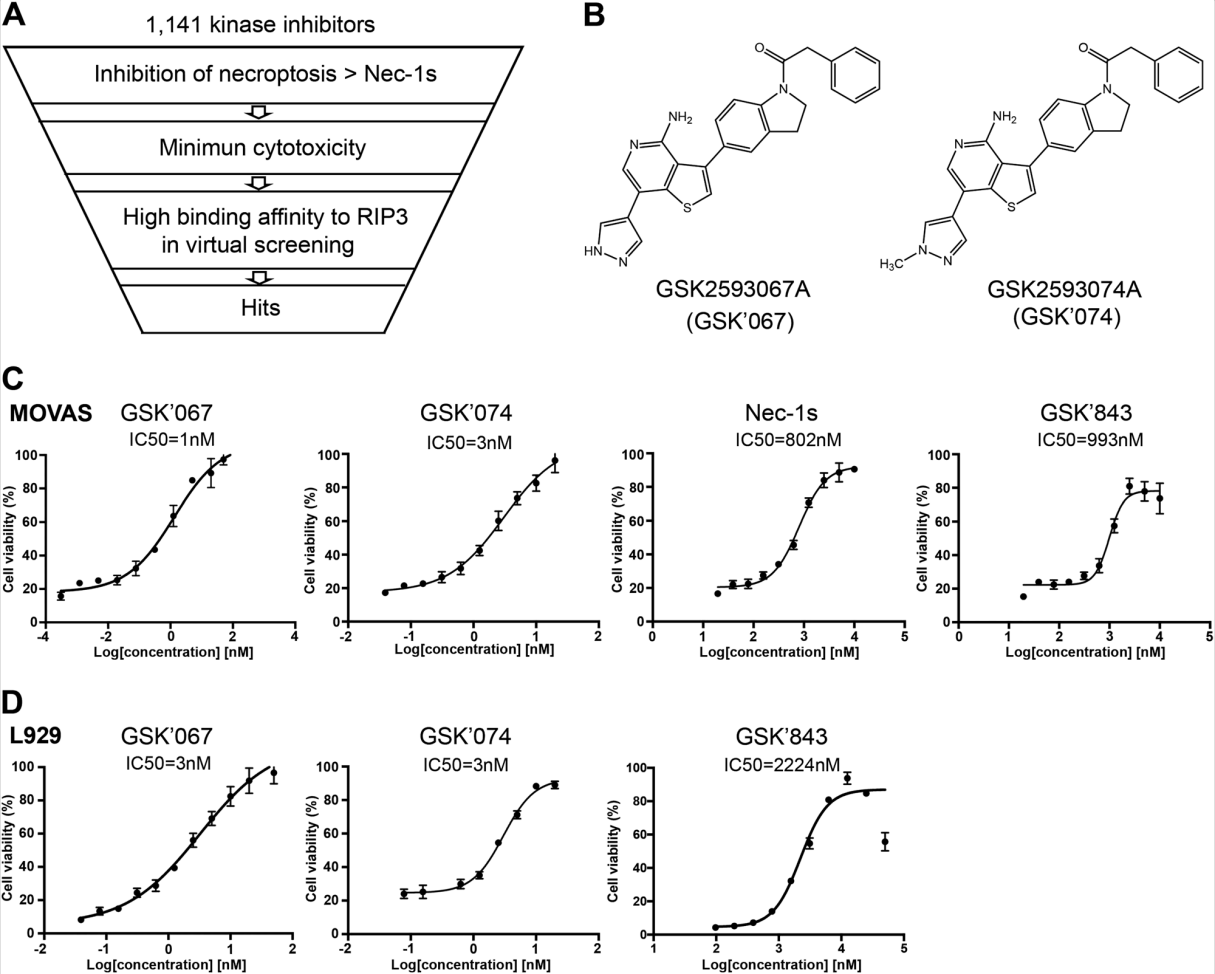
Identification of new necroptosis inhibitors. **a** Schematic overview of the drug screen workflow. **b** Chemical structure of GSK’067 and GSK’074. **c** Mouse smooth muscle cell line MOVAS were treated with 30 ng/ml TNFα plus 60 μM zVAD and indicated compounds for 6 h. Cell viability was detected by CellTiter-Glo. Data were normalized to DMSO treated control cells and presented as mean ± S.D. *n* = 3. **d** Mouse fibroblast cell line L929 were treated with 20 ng/ml TNFα plus 40 μM zVAD and GSK’067, GSK’074, or GSK’843 for 3 h. Cell viability was detected by CellTiter-Glo. Data were normalized to DMSO treated control cells and presented as mean ± S.D. of three independent experiments

To discover novel necroptosis inhibitors with high potency, safety, and selectivity, we performed library screen in three steps (Fig. 1a). In brief, the primary screen was conducted in a mouse aortic smooth muscle cell line (MOVAS) with combined TNFα (30 ng/ml) and a pan caspase inhibitor zVAD (60 μM) as a necroptosis induction protocol^14,16^. Cell viability was determined by CellTiter-Glo. Nec-1s (20 µM) was used as a reference compound for selection. Compounds (1 µM) that conferred more cellular protection than Nec-1s were advanced to secondary and tertiary screen for cytotoxicity and virtual binding to RIP3. A small cluster of structurally related compounds represented by GSK2593067A (GSK’067) and GSK2593074A (GSK’074) (Fig. 1b) met the selection criteria: (1) more potent necroptosis inhibition than Nec-1s; (2) minimum cytotoxicity (1 μM); and (3) low predicted docking energy to RIP3. In MOVAS, GSK’067 and GSK’074 dose-dependently protected cells against cell death (Fig. 1c). Comparing to Nec-1s and GSK’843 (a previously reported RIP3 inhibitor that shows structure similarity to GSK’067 and GSK’074), the new compounds showed lower IC50 and produced 10% more cell death inhibition than Nec-1s at 10 µM (Fig. 1c). Similarly, high potency of GSK’067 and GSK’074 was replicated in a mouse fibroblast cell line L929 cells (Fig. 1d). As GSK’067 and GSK’074 shared nearly identical chemical structures and cellular functions, we performed the rest of studies using only GSK’074.

**Fig. 2:**
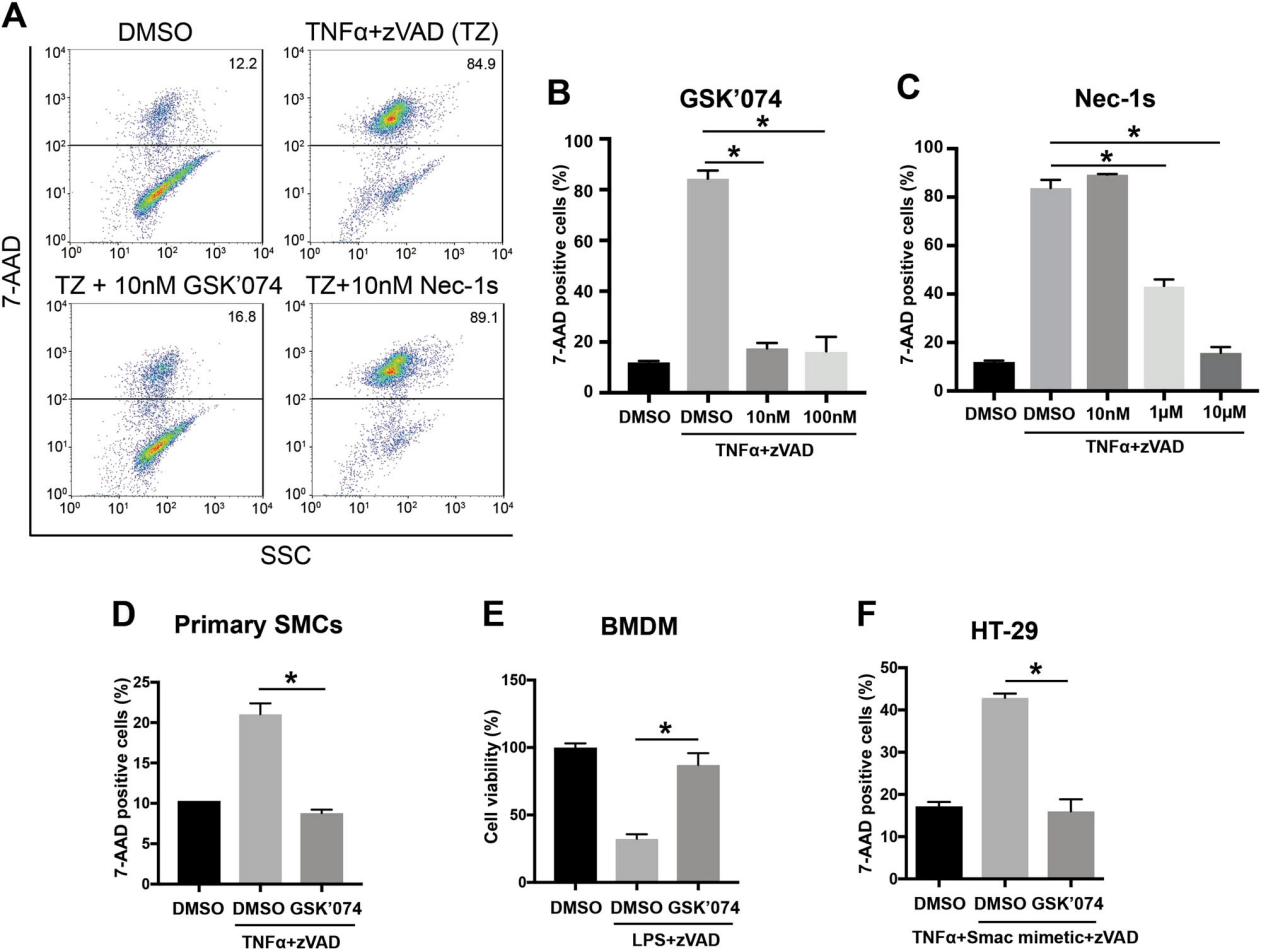
GSK’074 inhibits necroptosis in various cell types. **a–c** MOVAS cells were treated with 30 ng/ml TNFα plus 60 μM zVAD and compounds indicated for 6 h. Cells were then stained with 7-AAD and analyzed by flow cytometry. Necrotic cells were identified as 7-AAD^+^. **d** Mouse primary smooth muscle cells were treated with 100 ng/ml TNFα plus 60 μM zVAD and 10 nM GSK’074 for 24 h. Cells were then stained with 7-AAD and analyzed by flow cytometry. **e** Mouse bone marrow derived macrophages (BMDM) were treated with 10 ng/ml LPS plus 50 µM zVAD and 10 nM GSK’074 for 24 h. Cell viability was detected by CellTiter-Glo. **f** Human colorectal adenocarcinoma cell line HT-29 cells were treated with 20 ng/ml TNFα plus 200 nM Smac mimetic, 20 μM zVAD, and 10 nM GSK’074 for 24 h. Cells were then stained with 7-AAD and analyzed by flow cytometry. Data were presented as mean ± S.D. *n* = 3. *P < 0.05

Flow cytometric analyses following 7-AAD staining confirmed the anti-necroptosis property of GSK’074 (Necrotic cells: 7-AAD^+^, Fig. 2a). In MOVAS cells, 10 nM GSK’074 completely abolished necroptosis as evidenced by reducing 7-AAD^+^ population to basal level (untreated 11.99 ± 0.53%, DMSO 84.36 ± 3.19%, GSK’074 17.58 ± 2.04%), an effect comparable to what was produced by Nec-1s at 1000-fold higher dose (10 μM, 15.69 ± 2.45%; Fig. 2b, c). We next evaluated GSK’074 in primary aortic SMCs and macrophages, both play important roles in AAA development and progression. As shown in Fig. 2d, e, 10 nM GSK’074 conferred full protection to SMCs isolated from mouse aorta (7-AAD^+^ population: untreated 10.15 ± 0.21%, DMSO 21.05 ± 1.34%, GSK’074 8.81 ± 0.41%) and macrophages derived from mouse bone marrow (cell viability: untreated 100 ± 3.07%, DMSO 32.30 ± 3.38%, GSK’074 87.08 ± 8.76%), respectively. Moreover, we showed that GSK’074 was effective in inhibiting necroptosis of cells of human origin (human colorectal adenocarcinoma cell line HT-29 cells, 7-AAD^+^ population: untreated 17.17 ± 1.91%, DMSO 42.90 ± 1.41%, GSK’074 16.03 ± 4.86%; Fig. 2f).

### GSK’074 blocked necroptosis signaling

**Fig. 3:**
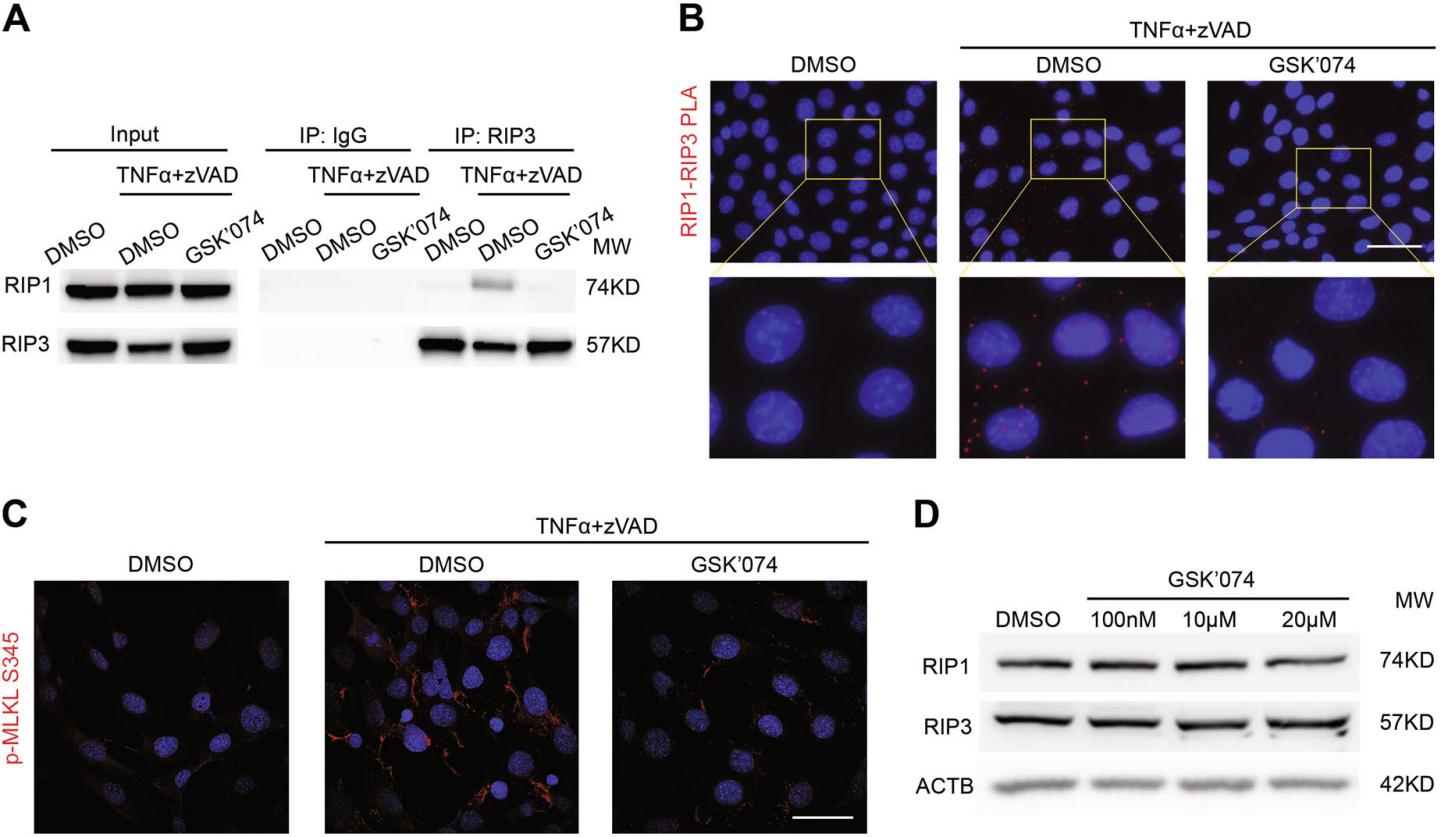
GSK’074 blocks necroptosis signaling pathway. **a**–**c** MOVAS cells were treated with 30 ng/ml TNFα plus 60 μM zVAD for 3 h in the presence or absence of GSK’074. RIP1 and RIP3 complex formation were detected by co-immunoprecipitation with anti-IgG or anti-RIP3 antibodies followed by immunoblot analysis with the indicated antibodies (**a**) or by in situ PLA assay (**b**). Scale bar = 50 µm. Cells were stained with anti-MLKL serine345 phosphorylation (p-MLKL S345) and representative pictures were shown (**c**). Scale bar = 50 µm. **d** MOVAS cells were treated with indicated concentrations of GSK’074 for 24 h, whole-cell lysates were subjected to immunoblot analysis with the indicated antibodies

Based on the structure similarity between GSK’074 and the previously established RIP3 inhibitor GSK’843, we postulated that the new inhibitors target RIP3 and thus inhibit RIP1-RIP3 containing necrosome formation that requires RIP3 kinase activity^26^. As expected, both MOVAS and L929 cells responded to necroptosis induction by forming RIP1-RIP3 complexes, detected by co-immunoprecipitation (co-IP) and proximity ligation assay (PLA). GSK’074 blocked RIP1-RIP3 complex formation (Fig. 3a, b, Supplementary Figure 1a, b). Furthermore, GSK’074 eliminated the RIP3-mediated mixed lineage kinase domain like pseudokinase (MLKL) serine345 phosphorylation without affecting protein levels of RIP1 and RIP3 (Fig. 3c, d and Supplementary Figure 1d).

### Dual inhibition of RIP1 and RIP3 by GSK’074

**Fig. 4:**
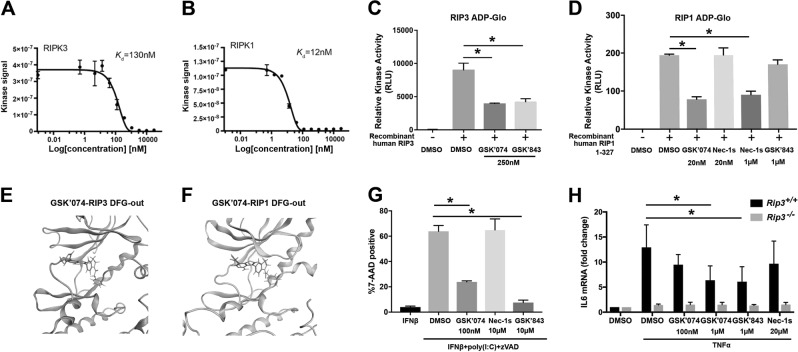
GSK’074 directly binds to RIP1 and RIP3. **a**, **b** The kinase domains of recombinant human RIP1 and RIP3 were used in the competitive binding assay of GSK’074. Data were presented as mean ± S.D. *n* = 2. **c**, **d** In vitro kinase activity was performed with recombinant RIP3 (**c**) or the kinase domain of RIP1 (1–327) (**d**). Levels of phosphorylation in the presence of compounds indicated were determined by ADP-Glo. Data were presented as mean ± S.D. *n* = 3. **e**, **f** Molecular docking of GSK’074 and kinase domain of human RIP3 (**e**) or RIP1 (**f**) in a DFG-out conformation. **g** L929 cells were primed with IFNβ (50 units/mL) for 24 h, then treated with 10 μg/ml poly(I:C) plus 40 μM zVAD and different concentrations of compounds indicated. Cells were stained with 7-AAD and analyzed by flow cytometry. Data represent mean ± S.D. of three independent experiments. **h** Mouse primary aortic smooth muscle cells were pretreated with indicated compounds for 2 h, followed by 10 ng/ml TNFα for additional 2 h. Levels of mRNA were determined by Real-time PCR. Data represent mean ± S.D. of three independent experiments. *P < 0.05

Using in vitro competitive binding assay, we demonstrated that GSK’074 bound to the kinase domain of recombinant human RIP3 with a Kd value of 130 nM (Fig. 4a). Unexpectedly, GSK’074 also displayed significant binding affinity to RIP1 (Kd = 12 nM) (Fig. 4b). In contrast, no detectable affinity was observed between GSK’074 and a nonrelated kinase (protein kinase C-delta or PKCδ; Kd > 30,000 nM). Consistently, in vitro kinase assay showed that GSK’074 inhibited kinase activity of both RIP1 and RIP3 (Fig. 4c, d). The dual inhibitory effects of GSK’074 on RIP1 and RIP3 set it apart from RIP3 inhibitor GSK’843 that showed no detectable inhibition on RIP1 (Fig. 4d). Of note, 20 nM of GSK’074 inhibited the RIP1 kinase activity to the same extent as 1 μM of Nec-1s did (Fig. 4d).

In the absence of a compound-protein co-crystal structure, we built homology models of human RIP1and RIP3 based on human B-Raf structures and probed how GSK’074 and GSK’843 might dock onto RIP3 or RIP1. The docking model predicted that GSK’074 bound preferably to the DFG-out homology models for both RIP1 and RIP3, suggesting that it was likely a type II kinase inhibitor that locked the kinase in an inactive conformation (Fig. 4e, f). GSK’074 was predicted to form typical H-bonds with the hinge backbone atoms of Lys 95 and Met 97 in RIP3 (Glu 93 and Met 95 in RIP1) and the pyrazole faced toward the solvent. In the DFG-out conformation, the benzyl portion of GSK’074 fit into the large hydrophobic pocket formed by Leu 73, Val 78, and Leu 131 in RIP3 (Leu 70, Val 75, and Leu 129 in RIP1). In contrast, the established RIP3 inhibitor GSK’843 preferred to bind the hinge region only and was therefore predicted to be a type I ATP competitive inhibitor (Supplementary Figure 1). The main interactions between GSK’843 and RIP3 were the canonical H-bonds with hinge residues, Lys 95 and Met 97. The benzothiazole ring of GSK’843 were packed by Leu67 and Thr 94, while like GSK’074, the central rings stacked with Phe 161, and the pyrazole ring was largely solvent exposed.

### GSK’074 inhibits RIP3-dependent RIP1-independent necroptosis and inflammation

Results from the in vitro binding studies and kinase assays raised a question whether GSK’074’s anti-necroptotic effect was solely dependent on RIP1. To test this, we turned to RIP1-independent necroptosis. Cells were primed with interferon-β (IFNβ, 50 units/mL) followed by adding poly (I:C) (10 μg/ml) and zVAD (40 μM) that was shown to cause Toll-like receptor 3-mediated necroptosis through the RIP1-independent TIR-domain-containing adapter-inducing interferon-β (TRIF)-RIP3- MLKL pathway^27^. In agreement with the literature, we showed that IFNβ plus poly(I:C) and zVAD (IPZ) significantly increased 7-AAD positive cells (DMSO control = 3.98 ± 0.56% vs. IPZ = 63.91 ± 3.21%). 100 nM GSK’074 or 10 μM GSK’843 significantly inhibited IPZ-induced necroptosis, reducing 7-AAD^+^ cells to 23.90 ± 0.56% and 7.55 ± 1.36%, respectively (Fig. 4g and Supplementary Figure 3). In line with the literature^25,27^, inhibition of RIP1 had no significant effect on the TLR3-mediated necroptosis.

Low-dose TNFα-induced cytokine expression in vascular SMCs is another established RIP3-dependent, RIP1-independent cellular event^14,16^. As shown in Fig. 4h, GSK’074, as well as the RIP3 inhibitor GSK’843, but not Nec-1s, significantly repressed expression of IL6 triggered by 10 ng/ml of TNFα. The critical role of RIP3 in inflammation was further demonstrated by the profound difference in the TNFα response of *Rip3*^*+/+*^ and *Rip3*^−/−^ aortic SMCs. The insensitivity of *Rip3*^−/−^ primary cells to GSK’074 asserts that the new inhibitor functions through RIP3.

### GSK’074 lacks pro-apoptosis property

**Fig. 5:**
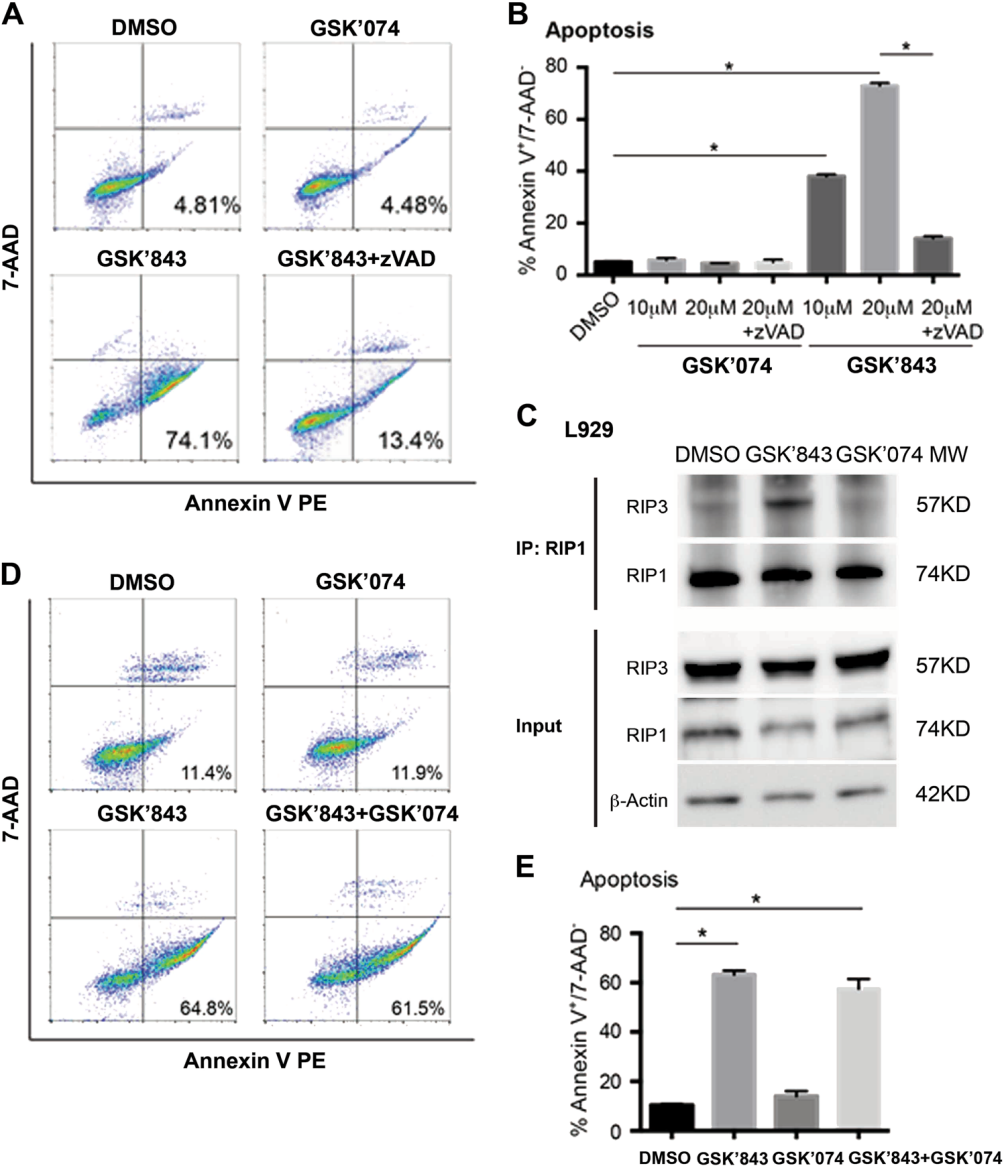
GSK’074 does not induce apoptosis. **a**, **b**, **d**, **e** L929 cells were treated with indicated compounds for 24 h, then stained with Annexin V-PE and 7-AAD and analyzed by flow cytometry. Apoptotic cells were identified as PE Annexin V^+^/7-AAD^−^. **c** L929 cells were treated with 10 μM GSK’843 or GSK’074 for 24 h, cell lysates were immunoprecipitated with anti-RIP1 antibody followed by immunoblot analysis with the indicated antibodies. *P < 0.05

High concentrations (≥3 μM) of RIP3-selective inhibitors including GSK’840, GSK’843, and GSK’872 cause apoptosis through a mechanism that does not require kinase activity of RIP3 or RIP1^25^. Consistently, we also showed that GSK’843 dose-dependently caused apoptosis (Annexin V^+^/7-AAD^−^) that was abolished by a pan-caspase inhibitor zVAD (Fig. 5a, b). In addition, we detected the pro-apoptotic ripoptosome-like death complex in cells treated with 10 μM GSK’843 (Fig. 5c). In a sharp contrast, 10 μM GSK’074 did not increase cell apoptosis as compared to control or led to formation of the pro-apoptotic complex (Fig. 5a–c). We next tested whether GSK’074 had anti-apoptosis properties. As shown in Fig. 5d, e, GSK’074 did not block apoptosis induced by GSK’843. Moreover, GSK’074 failed to protect MOVAS cells from the endoplasmic reticulum (ER) stress-dependent apoptotic death induced by tunicamycin (Supplementary Figure 2a). Due to the structural relationship between GSK’074 and a previously reported protein kinase R-like endoplasmic reticulum kinase (PERK) inhibitor GSK2606414 (GSK’414)^28^, we tested whether PERK participates in necroptosis signaling. As shown in Supplementary Fig. 2b, knocking down of PERK in L929 cells had no significant effect on necroptosis.

Taken together, GSK’074 differs from previously reported RIP3 inhibitors in three important aspects: (1) it is capable of binding to both RIP3 and RIP1 and inhibits their kinase activities, (2) it does not drive RIP3 to a kinase-independent pro-apoptotic configuration, and (3) it may work as a type II kinase inhibitor. Therefore, GSK’074 represents a new class of necroptosis inhibitors.

**Fig. 6:**
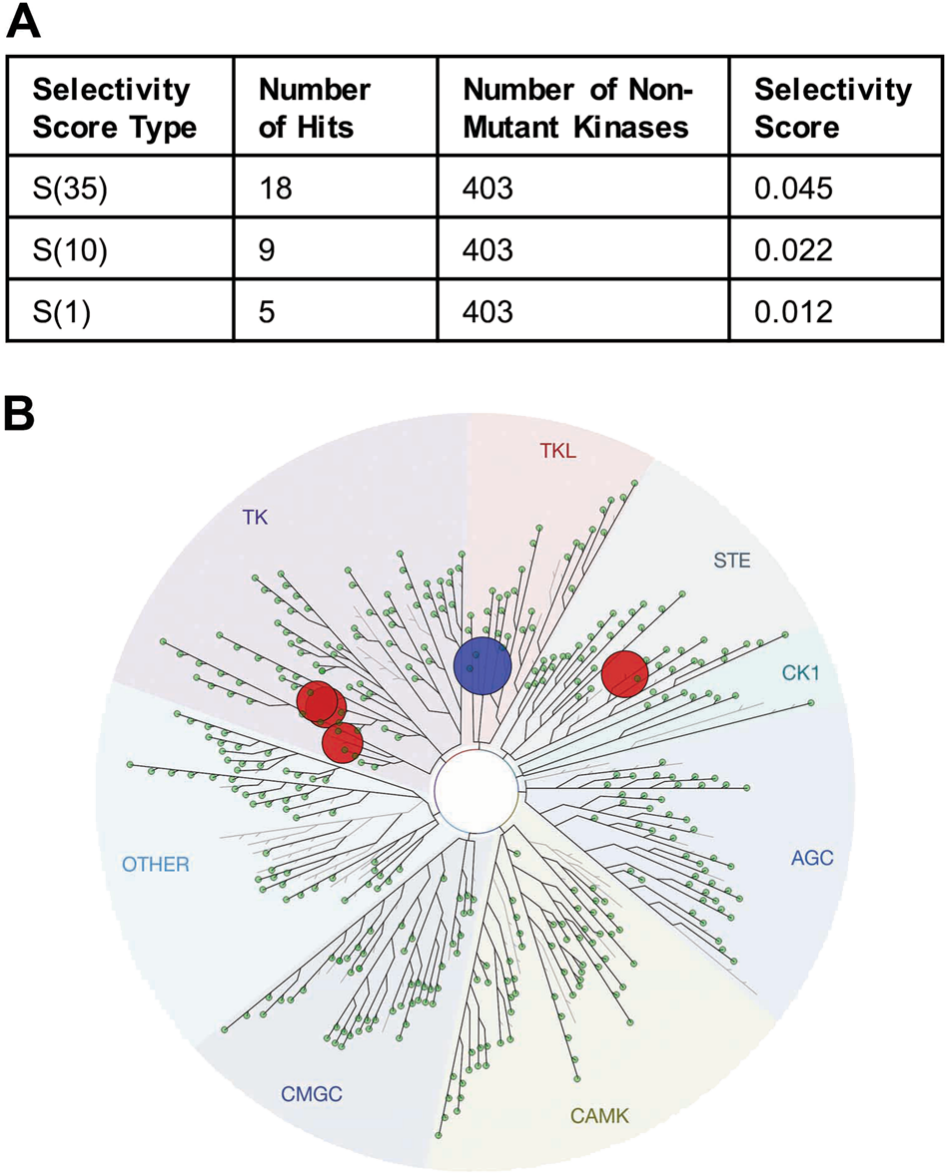
Selectivity profile of GSK’074. Binding of GSK’074 (100 nM) against 468 human kinases (403 non-mutant kinases, 65 mutant kinases) was assessed by DiscoveRx KINOME*scan*™. **a** Selectivity scores of GSK’074. Selectivity score was defined as the number of non-mutant kinases bound to GSK’074 <35% of control (S(35)), 10% of control (S(10)), or 1% of control (S(1)). **b** TREEspot map of kinases potentially targeted by GSK’074. Binding with RIP1 (0% of control) was shown by the blue circle, red circles indicated other kinases that displayed similar binding as RIP1 (S(1))

Next, we investigated the selectivity profile of GSK’074 against a panel of 468 kinases, including 403 non-mutant kinases using the KINOME*scan*™ profiling service. At a concentration of 100 nM, GSK’074 was found to be highly selective for RIP1 (0% of control). Supplementary Table 1 showed the kinases that bound to GSK’074 <35% of control. With a selectivity score S(1) of 0.012 (representing a hit rate of 5 out of 403 non-mutant kinases at 1% of control or below), GSK’074 also bound to four other kinases (KIT, MEK5, CSF1R, and EPHB6) with affinities that were close to its binding to RIP1 (<1% of control; Fig. 6). KIT, MEK5, CSF1R, or EPHB6 had no established roles in cell death and their expression levels in SMCs, with the exception of MEK5, were negligible (Supplementary Figure 3).

### GSK’074 inhibits necrosis and inflammation in mouse models of aortic aneurysm

**Fig. 7:**
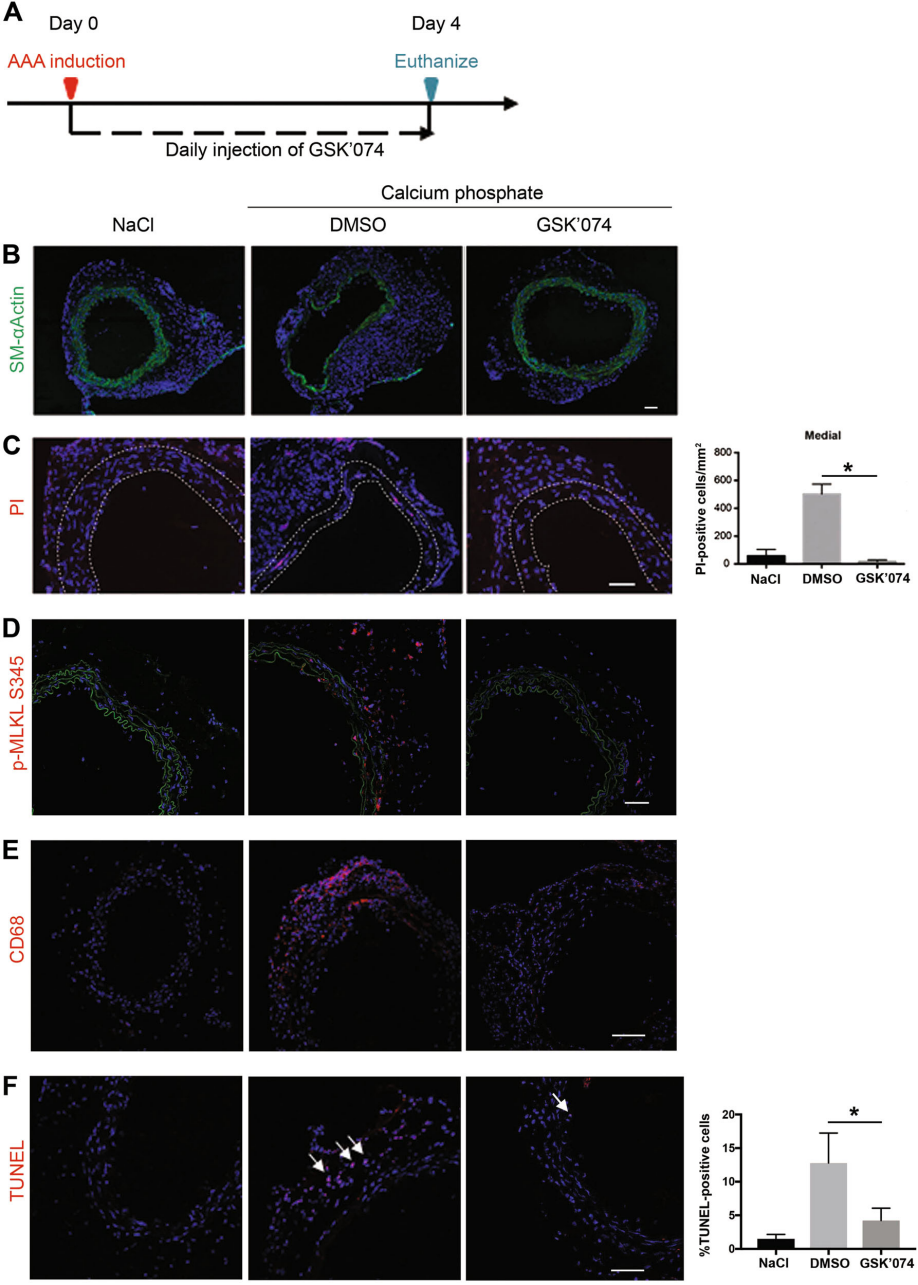
GSK’074 attenuates cell death and inflammation in calcium phosphate injured aortae. **a** Experimental design of the calcium phosphate aneurysm model. Mice were treated with 200 µl vehicle (8% DMSO) or GSK’074 at 0.93 mg/kg/day immediately following aneurysm induction. Three male mice were in each group. Mice were euthanized 4 days after. **b**, **e** Representative photographs of immunostaining for markers smooth muscle cell (SM-αActin) or macrophages (CD68). Scale bar = 50 μm. **c** Mice were injected with propidium iodide (PI) 2 h before euthanization. DAPI was used to stain nuclei. Quantification of PI-positive cells in the medial layer (indicated by white dashed line) was shown on the right. Scale bar = 50 μm. **d** Cross-sections of aneurysm prone tissue were immunostained with anti-MLKL serine345 phosphorylation (p-MLKL S345) as a marker of necroptosis. Scale bar = 50 μm. **f** Representative photographs of terminal deoxynucleotidyl transferase dUTP nick end labeling (TUNEL) in aortic cross-sections. Quantification of TUNEL-positive cells percentage was shown on the right. Scale bar = 50 μm. *P < 0.05

**Fig. 8:**
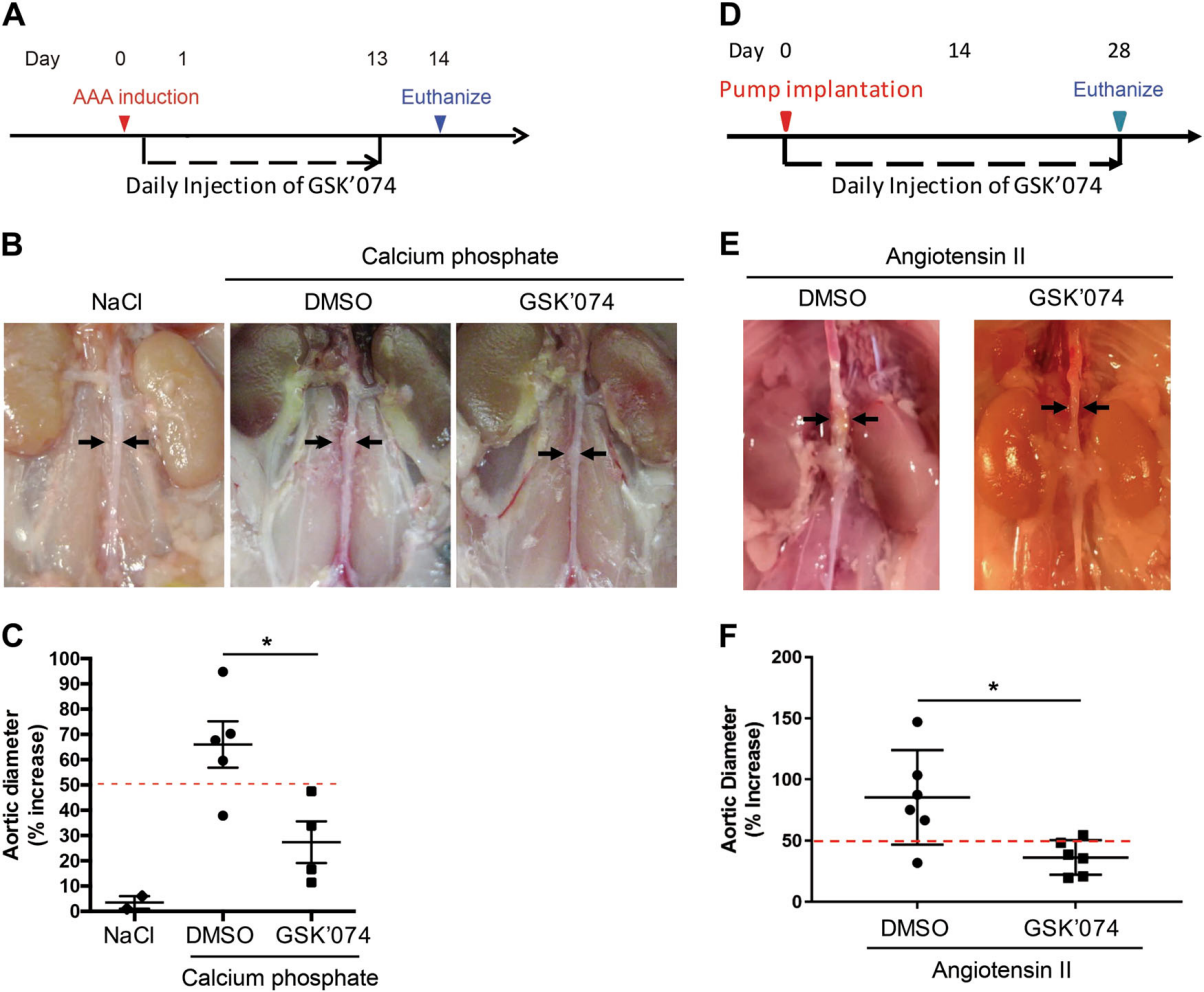
GSK’074 inhibits aneurysm formation in mouse models of aneurysms. **a**, **d** Experimental design. Abdominal aortic aneurysm was induced in male C57BL/6J mice (8–10 weeks) by the calcium phosphate aneurysm model (**a**) or in *Apoe*^*−/−*^ female mice (9–10 months) by the Angiotensin II infusion (1000 ng/kg/min) (**d**). 200 µl vehicle (8% DMSO) or GSK’074 at 0.93 mg/kg/day was administered daily via IP. Mice were euthanized 14 (**a**) or 28 days (**d**) after aneurysm induction. **b**, **e** Representative photos of perfused abdominal aortae with indicated treatments. Arrows indicate aneurysm formation. **c**, **f** Percentage increase of maximal external aortic diameter. An AAA is defined as a percentage increase in aortic diameter ≥50% (red dashed line) compared to the aortic diameter before calcium phosphate treatment (**c**) or compared to the external diameter of infrarenal diameter (**f**). Data were presented as mean ± S.D. *P < 0.05

With minimum cytotoxicity and high anti-necroptotic anti-inflammatory properties, GSK’074 and its analogs are suitable for in vivo applications. Furthermore, the half-life of GSK’074 in liver microsome was ~1 h, similar to that of Nec-1s, the improved version of Nec-1^29^. Based on the formerly described dose response study in which GSK’074 inhibited SMC necroptosis with an IC50 that was ~0.3% of that of Nec-1 s, we selected 0.93 mg/kg/day (2 μmol/kg/day) to test this new class of inhibitors in mouse models of aortic aneurysm. We recently reported that 1.6 mg/kg/day of Nec-1s blocked aneurysm formation^16^. GSK’074 or its solvent 8% DMSO was administrated via daily intraperitoneal (IP) injection, starting immediately after the induction of aneurysm by calcium phosphate, a modification of the calcium chloride aneurysm model^30^. Mice were euthanized on Days 4 and 14 post-surgery for evaluation of cellular events and aortic dilatation, respectively (Fig. 7a and Fig. 8a). The pathophysiology of an aneurysm was confirmed by diminished levels of smooth muscle-αActin (SM-αActin) and increased inflammatory infiltration in the aneurysm prone tissues 4 days after surgery. GSK’074 treatment preserved expression of SM-αActin in calcium phosphate treated arteries (Fig. 7b). We used propidium iodide **(**PI) to stain necrotic cells as previously reported^14^. PI was IP injected 2 h prior to euthanization. Phospho-MLKL was examined as a histological marker of necroptosis. The aneurysm prone aortic tissues of the DMSO-treated mice contained PI-positive necrotic cells, elevated levels of phospho-MLKL, as well as terminal deoxynucleotidyl transferase dUTP nick end labeling (TUNEL)-positive apoptotic cells and CD68-positive macrophages (Fig. 7c–f). GSK’074 decreased the number of necrotic cells, MLKL phosphorylation, as well as apoptotic cells and macrophages. (Fig. 7c–f). Together, these results demonstrated that GSK’074 treatment not only inhibited necroptosis following aneurysm induction but also attenuated apoptosis and macrophage infiltration.

Next, we evaluated aneurysm formation by measuring external diameter of inferrenal aorta. Compared to the sodium chloride group, aortas treated with calcium phosphate showed visible dilatation (Fig. 8b, c). The aortic dilatation in 4 out of 5 DMSO-treated mice reached the aneurysm threshold (Fig. 8b, c), which was defined as a 50% or more increase in the maximal external aortic diameter compared to that measured before calcium phosphate application^30^. In contrast, none of the four GSK’074-treated mice developed aneurysm. In addition to the reduction of aneurysm incidence, GSK’074 significantly decreased the extent of aortic expansion (DMSO 66.06 ± 9.17% vs GSK’074 27.36 ± 8.25%; *P* < 0.05; Fig. 8b, c). To assess the therapeutic potential of our inhibitor in an experimental setting that more closely resembles clinical situations and in females (the above described calcium phosphate model was conducted in young male mice), we next induced aortic aneurysm formation with Angiotensin II (Ang II) infusion in 9-month-old apolipoprotein E–deficient (*Apoe*^*−/−*^) female mice. GSK’074 (0.93 mg/kg/day) or 8% DMSO was administrated daily via IP injection to *Apoe*^−/−^ mice immediately following pump implantation (Fig. 8d). Compared to the DMSO group, GSK’074-treated mice showed significantly alleviated aneurysm formation (Fig. 8e, f), reflected by a much smaller aortic dilatation (DMSO 85.39 ± 15.76% vs GSK’074 36.28 ± 5.76%; *P* < 0.05) as well as a reduced AAA incidence (from 83.3 to 16.7%; Fig. 8e, f).

## Discussion

In this study, we identified a novel class of necroptosis inhibitor represented by GSK’074 that completely rescued cells from necroptosis under different stimuli in both human and murine cells at IC50 ~3 nM. Several necroptosis inhibitors have been previously reported; however, the therapeutic potentials of the existing inhibitors are limited by low potency (Nec-1 inhibits necroptosis in the micromolar range^29^) or poor selectivity (in the case of ponatinib^31^). PN10, generated by fusing ponatinib with Nec-1s, shows high in vitro efficacy and selectivity, but its high molecular weight hinders compound absorption and tissue distribution^32^. Similar limitations are also reported of other RIP1 inhibitors such as GSK’481, GSK’963, cdp27, and GSK’772^33–36^. Relatively, fewer RIP3 inhibitors are available. Besides GSK’843, GSK’840, and GSK’872 reported by Mandal et al.^25^, another RIP3 inhibitor GW’39B shares a similar structural core as GSK’872. However, it remains unclear whether GW’39B causes apoptosis as GSK’872^37^. B-Raf inhibitor dabrafenib binds to RIP3 in a cell-free system, but the low potency hinders its application in treating necroptosis-involving diseases^38,39^. Considering these various limitations of known RIP1 and RIP3 inhibitors, our discovery of a new class of safe and potent small compounds with abilities to attenuate necroptosis as well as cytokine expression is of significance.

We assert that GSK’074 and its analogs are dual RIP1/RIP3 inhibitors based on multiple lines of evidence including binding to recombinant RIP1 and RIP3, inhibiting kinase activities of either kinases, and blocking cellular functions that depend on both kinases or RIP3 only. Results from our homology modeling support the notion of dual inhibitors. In addition, molecular docking results suggest that GSK’074 binds to RIP1 and RIP3 on a DFG-out conformation while GSK’843 binds to RIP3 as an ATP competitive inhibitor. Interestingly, dabrafenib was also predicted to interact with RIP3 as an ATP competitive inhibitor by a similar computation model^38^. Typically, a DFG-out conformation within activation loop indicates an inactive state of a protein kinase while the DFG-in conformation is associated an active state^40^. Inhibitors that lock protein kinases in a DFG-out conformation are referred as type II inhibitors whereas inhibitors that target the ATP-site of the kinase in its active, DFG-in state, are referred as type I inhibitors. Type II inhibitors possess several advantages over type I inhibitors, including improved kinase selectivity and slower off-rates^41^. Since the majority of kinase inhibitors target the ATP-site, GSK’074 is likely to be the first necroptosis inhibitor capable of binding to RIP3 as a type II kinase inhibitor.

It is unclear why the new inhibitors do not cause apoptosis even though they share a similar core chemical structure with the pro-apoptotic RIP3 inhibitor GSK’843. We think apoptosis is not an inherited outcome of RIP3-kinase inhibition because not all kinase dead RIP3 mutants cause apoptosis. While D161N, D161G, D143N, and K51A all lead to disruption of the kinase activity of RIP3, only D161N mutant activates the apoptotic signaling^25^. We attribute the lack of cytotoxicity of new RIP3 inhibitors to their unique interaction with the RIP3 molecule. Although this notion remains to be experimentally tested, our data demonstrate that GSK’074 and analogs do not possess anti-apoptotic properties. GSK’074 had no effect on apoptosis caused by GSK’843 or by tunicamycin that induces ER stress via PERK-ATF4-CHOP signaling^42^. Of note, the PERK inhibitor GSK’414 was recently found to inhibit necroptosis through RIP1 but independent of PERK^43^. GSK’414 was included in one of the libraries that we screened and showed anti-necroptosis properties during the primary screen. However, GSK’414 did not make to our final list of positive hits due to low maximum effect and a relative poor predicted binding affinity to RIP3 (Supplementary Figure 4). Although PERK is not in the library of kinases used in our KINOME*scan*™, PERK is unlikely to underlie the anti-necroptotic effect of GSK’074 because knocking down PERK was inconsequential to necroptosis.

In theory, being able to inhibit both RIP1 and RIP3 allows GSK’074 and analogs to protect cells from a broader range of stimuli. In cardiomyocyte, ischemia-reperfusion induced necroptosis is mediated by RIP3- CaMKII-mPTP but independent of RIP1 and MLKL^44^. Besides RIP1, RIP3 can also interact with other factors containing RHIM domain, such as TRIF and DAI, or only RIP3 dimerization, to activate necroptosis^3,27,45^. Our previous studies also demonstrated that in SMCs, low dose of TNFα induces inflammatory cytokine expression via a RIP3-dependent, RIP1-independent mechanism^14,16^. In this study, 100 nM GSK’074 ameliorated IFNβ plus poly(I:C) and zVAD induced RIP1-insensitive necrosis in L929 cells, and IL6 expression in SMCs caused by low dose TNFα. These data ratify RIP3 as a biological target of the new inhibitors.

AAA is a life-threatening disease with no available pharmacological therapy. This progressive degenerative vascular disease is characterized by depletion of smooth muscle cells, inflammation, negative extracellular matrix remodeling, and progressive expansion of the aorta. In this study, daily IP injection of GSK’074 significantly blocked aneurysm growth in two mouse models that induced aortic aneurysm through distinct mechanisms. Immunostaining results showed that GSK’074 reduced necrosis in aneurysm-prone aortae, especially in medial layer. Apoptosis in adventitia was also alleviated, maybe due to reduced macrophage infiltration, as we previously found that RIP1 was involved in macrophage-induced SMC apoptosis^46^. There were only few apoptotic cells detected in medial layer, which is likely due to the late time point when tissues were examined. Apoptosis typically peaks soon after aneurysm induction^47^. Although mouse studies do not reliably predict the outcome of clinical trial, the biochemical and pharmacological properties of our new inhibitors make them good candidates for further therapeutic development.

In summary, we identified a new class of necroptosis inhibitors represented by GSK’074 which inhibited RIP1 and RIP3 as type II kinase inhibitors. GSK’074 displayed high potency in both human and murine cells. The definite benefits provided by GSK’074 in mice aneurysm models of both male and female were encouraging and warrant further pharmacological studies in models of aortic aneurysm as well as other human diseases involving necroptosis and inflammation.

## Materials and methods

### General materials

Dulbecco's Modified Eagle Medium (DMEM) was purchased from Gibco (Life Technologies, Carlsbad, CA), recombinant mouse TNFα from R&D Systems (Minneapolis, MN), Z-VAD-FMK (zVAD) from Bachem (Torrance, CA), and 7-Cl-O-Nec-1 (Necrostatin-1s, Nec-1s) from EMD Millipore (Burlington, MA). Primary antibodies including anti-MLKL, anti-MLKL (phospho S345, ab196436), anti-SM-αActin, and anti-CD68 were purchased from Abcam (Cambridge, MA), anti-RIP1 was purchased from BD Biosciences (San Jose, CA), anti-RIP3 from ProSci (Poway, CA), anti-β-Actin (ACTB) from Sigma-Aldrich (St. Louis, MO). Fluorophore-conjugated secondary antibodies and 4′,6-diamidino-2-phenylindole dihydrochloride (DAPI) were purchased from Molecular Probes (Life Technologies, Carlsbad, CA). Horseradish Peroxidase (HRP)-conjugated antibodies were purchased from Bio-Rad (Hercules, CA). In situ Cell Death Detection Kit was purchased from Roche Applied Science (Indianapolis, IN). Other chemicals and reagents if not specified were purchased from Sigma-Aldrich (St. Louis, MO).

### Cell culture

Mouse aortic smooth muscle cell line MOVAS cells, mouse fibroblast cell line L929 cells and human colorectal adenocarcinoma cell line HT-29 cells were obtained from American Type Culture Collection (ATCC, Manassas, VA) and grown as recommended in DMEM modified containing 4.5 g/L d-Glucose (Life Technologies, Carlsbad, CA) supplemented with 10% fetal bovine serum (FBS), 100 U/mL penicillin, and 100 U/mL streptomycin. Primary mouse aortic SMCs were isolated from the abdominal aorta as described previously^14^. Smooth muscle identity was validated by expression of smooth muscle protein 22-α (SM22α) and SM-αActin, while lacking endothelial cell marker CD31 or fibroblast marker ER-TR7. Cells between three and seven passages were used. Bone marrow derived macrophages were isolated and cultured as described before^48^. In brief, bone marrow was flushed from long bones and washed with PBS, then suspended in DMEM supplemented with 10% L-cell conditioned media (LCCM). LCCM media was collected from L929 cells cultured in T-75 cm^2^ filter cap flasks in DMEM for 10 days and filtered through 0.2 μm. Seven days after harvest, all non-adherent cells were removed, and remaining cells were split in 96-well plates.

### Small chemical library screen

Chemical screening was conducted in UW Small Molecule Screening Facility (hts.wisc.edu). MOVAS cells, grown in 384-well plates, were induced to undergo necroptosis with 30 ng/ml TNFα and 60 μM zVAD. Compounds (1141) from 3 kinase inhibitor libraries (GSK 2014 set 1, GSK 2014 set 2, and Selleck-1) were delivered into each well at a final concentration of 1 μM. Cell viability was determined by Cell Titer-Glo assay. The lead compound GSK’074 was synthesized according to published synthesis scheme of similar compounds^28^.

### Mice

C57BL/6J and *Apoe*^−/−^ mice were purchased from The Jackson Laboratory (Bar Harbor, Maine). Male mice age 3-month-old or female mice age 9-month-old were used for experiments. All animal experiments were approved by the Institutional Animal Care and Use Committee at the University of Wisconsin–Madison (protocol number: M005792). The procedures were carried out in accordance with the approved guidelines.

### Calcium phosphate-induced murine AAA

3-month-old C57BL/6J male mice were subjected to calcium phosphate treatment as described^30^. In brief, the infrarenal region of the abdominal aorta was isolated following a midline incision. A small piece of gauze soaked in 0.5 M calcium chloride was applied perivascularly for 10 min, followed by application of phosphate-buffered saline (PBS)-soaked gauze for 5 min. The control mice received 0.5 M sodium chloride-soaked gauze for 10 min followed by PBS soaked gauze for 5 min. The external diameter of the largest portion of an abdominal aorta was measured with a digital caliper (VWR Scientific, West Chester, PA) prior to treatment and at the time of tissue harvest. Aneurysm incidence was defined as an increase of 50% or greater in the external width of the abdominal aorta compared with that before calcium phosphate treatment.

### Angiotensin II-induced murine AAA

Female *Apoe−/−* mice were aged to 9-month old under regular diet. Angiotensin II (Ang II; 1000 ng/kg/min. Sigma-Aldrich A9525) was administered subcutaneously by Alzet osmotic minipumps (Alzet model 2004, Cupertino, Calif) for 28 days^16,49^. Mice were randomly grouped to receive either GSK’074 (0.93 mg/kg/day) or 8% DMSO via daily IP injection immediately following minipump implantation. The external aortic diameter was measured at the region showing maximum dilation with a digital caliper. Aneurysm incidence was defined as an increase of 50% or greater in the external width of the suprarenal aorta as compared with that of the infrarenal region.

### RNA isolation and real-time PCR

Total RNA was extracted from cultured cells using Trizol reagent (Life Technologies, Carlsbad, CA) according to the manufacturer's protocols. Two micrograms of total RNA was used for the first-strand cDNA synthesis (Applied Biosystems, Carlsbad, CA). Real-time PCR (RT-PCR) was carried out using the 7500 Fast Real-Time PCR System (Applied Biosystems, Carlsbad, CA). Each cDNA template was amplified in triplicate using SYBR Green PCR Master Mix (Applied Biosystems, Carlsbad, CA) with gene specific primers. Primers for RT-PCR were QuantiTect Primers purchased from Qiagen (Valencia, CA). The relative mRNA levels were calculated using the 2−ΔΔCT method. β-Actin was used as the endogenous control.

### Flow cytometric analysis

Cell death was evaluated by using an Annexin V-PE/7-AAD staining Kit (BD Biosciences, San Jose, CA). Cultures were rinsed with ice-cold PBS and incubated with accutase (Life Technologies, Carlsbad, CA) at 37 °C for 2 min. The detached cells were collected by centrifugation (2000 rpm, 5 min). Cell pellets were further washed twice with ice-cold PBS and resuspended in 100 μl 1× binding buffer from the Annexin V-PE/7-AAD staining Kit. Five microlitre of Annexin V-PE and 5 μl of 7-AAD were added to the cells and incubated at room temperature for 15 min. After incubation, 400 μl binding buffer was added to each sample. Cells were analyzed using a Becton Dickinson Biosciences FACSCalibur (BD Biosciences, San Jose, CA).

### In vivo propidium iodide staining

In vivo cell necrosis was examined by IP injection of propidium iodide (PI) as previously described^14^. PI (15 mg/kg body weight) was administered to mice through IP injection. Two hours after PI administration, mice were euthanized and perfusion fixed with 4% formaldehyde. Cryosections of 6-μm-thick were cut. Mounting medium with DAPI was applied before fluorescence microscopy examination. Staining was immediately visualized with a Nikon Eclipse Ti inverted microscope system and digital images were acquired using a Nikon DS-Ri1 digital camera.

Semi-quantification analysis of PI-positive cells in diseased tissues were performed as previously described^14,16^. PI-positive cells were counted in a blind fashion. The area of tunica media was measured with ImageJ (National Institute of Health, Bethesda, MD). At least three non-serial cross-sections per aorta were analyzed for each mouse (*n* = 3 aortae per treatment).

### Immunohistochemistry

Aortas were perfusion-fixed with a mixture of 4% formaldehyde in PBS under physiological pressure in order to preserve the structural morphology. Tissues were imbedded in O.C.T. Compound (Sakura Tissue Tek, Netherlands) and sectioned to 6 μm thickness using a Leica CM3050S cryostat. Tissue sections were stained and analyzed as described previously^16^.

Terminal deoxynucleotidyl transferase dUTP nick end labeling (TUNEL) and semi-quantification analysis of TUNEL-positive cells in aneurysmal tissues were performed as previously described^16^. TUNEL-positive cells were counted in a blind fashion. The area of aorta was measured with ImageJ (National Institute of Health, Bethesda, MD). At least three non-serial cross-sections per aorta were analyzed (*n* = 3 aortae per treatment).

### Immunoblotting

Cells were lysed in RIPA buffer (Sigma-Aldrich, St. Louis, MO) containing protease and phosphatase inhibitors (Halt Cocktail, Thermo Scientific, Rockford, IL) (floating cells in the medium were collected by centrifugation (2000 rpm, 5 min) and combined with attached cells). Equal amounts of protein extract were loaded and separated by SDS-PAGE and then transferred to polyvinylidene fluoride (PVDF) membranes. The membranes were blocked for 60 min at room temperature with 5% skim milk in Tris-buffered saline plus 0.05% Tween 20 (TBST), and then incubated with primary antibodies overnight at 4 °C, followed by HRP-labeled secondary antibodies. Labeled proteins were visualized with an enhanced chemiluminescence system (PerkinElmer-cetus, Boston, MA) and ImageQuant LAS 4000 Mini (GE Healthcare Bio-Sciences, P.O. Pittsburgh, PA). For quantification, optical densities of proteins were determined by ImageJ (National Institute of Health, Bethesda, MD).

### Co-immunoprecipitation

Cells were lysed in Pierce IP Lysis Buffer (Pierce, Waltham, MA; floating cells in the medium were collected by centrifugation (2000 rpm, 5 min) and combined with attached cells), then co-immunoprecipitation experiments were performed using the SureBeads magnetic beads (Biorad, Hercules, CA) according to the manufacturer’s protocol. In brief, Protein A magnetic beads were washed in PBST then incubated with anti-RIP3 antibody (ProSci, Poway, CA) or its isotype control for 30 min at room temperature. Beads were magnetized and washed 3 times with PBST, then incubated with cell lysate for 1 h at room temperature. After incubation, beads were washed 3 times with PBST, and immunoprecipitated proteins were eluted in 1x Laemmli buffer and subjected to immunoblotting.

### Kinase activity assay

Kinase activities of recombinant human RIP3 or RIP1 (1–375) kinase domain were determined using ADP-Glo (Promega, Madison, WI), which measures the conversion of ATP to ADP as previously described^34^. Test compounds of indicated concentrations in assay buffer (50 mM Hepes pH 7.5, 50 mM NaCl, 30 mM MgCl_2_, 1 mM DTT, 0.02% CHAPS, 0.5 mg/mL BSA) was combined with 25 nM kinase and substrate in a 384-well low flange white plate (Corning, Corning, NY). Following a 1-hour incubation, 150 µM ATP was added to the plate for additional 5 h. The reaction was terminated by sequential addition of the ADP-Glo Reagent and ADP-Glo Detection solution. The luminescence was measured on a FlexStation 3 (Molecular Devices, San Jose, CA) and normalized to no kinase control.

### In situ proximity ligation assay

An in situ proximity ligation assay (PLA) was performed to detect protein–protein interactions using a Duolink in situ fluorescence kit according to the manufacturer’s protocol (Olink Bioscience, Uppsala, Sweden). In brief, cells were washed with PBS (floating cells were washed away), then fixed with 4% paraformaldehyde at room temperature for 10 min followed by cell membrane permeabilization with 0.2% Triton X-100 in PBS for 10 min. Tissue sections were fixed for 10 min in cold acetone. The slides were washed three times with PBS, blocked for 1 h at room temperature with 5% BSA and normal donkey serum in PBS, and incubated with the indicated antibody pairs overnight at 4 °C. Oligonucleotide-conjugated secondary antibodies (PLA probe MINUS and PLA probe PLUS) against each of the primary antibodies were applied, and ligation and amplification were carried out to produce rolling circle products. These products were detected with fluorescently labeled oligonucleotides, and the sections were counterstained using Duolink Mounting Medium with DAPI. Samples were examined using a Nikon microscope (Melville, NY).

### Molecular docking

Homology models of RIP1 and RIP3 were built based on human B-Raf structures PDB:4FK3 for DFG-in and PDB:3IDP for DFG-out. Initial models were built using MOE (v2016.08 Chemical Computing Group, Montreal) and were then minimized with AMBER10 force field. Ligand docking was done using HYBRID (v3.2.0, OpenEye Scientific, Santa Fe) in the OE Docking tools, with up to 1000 conformations of each ligand and using the MMFF charge model. All ligands were docking into both DFG-in and DFG-out homology models of both RIP1 and RIP3.

### KINOME*scan*™ analysis

The selectivity of GSK’074 was evaluated using KINOMEscan™ profiling service (DiscoveRx Corporation, Fremont, CA). In brief, an in vitro competition binding assay was used to evaluate GSK’074 specificity at a concentration of 100 nM against 468 human kinases including 403 non-mutant kinases and 65 mutant kinases, as described previously^33,50^. KINOME*scan*™ was also used to determine binding constants (Kd) for GSK’074 against RIP1 and RIP3. In brief, an 11-point threefold serial dilution of GSK’074 was prepared in 100% DMSO at 100× final test concentration and subsequently diluted to 1× in the assay buffer. Kds were calculated with a standard dose-response curve using the Hill equation. Curves were fitted using a non-linear least square fit with the Levenberg–Marquardt algorithm.

### Statistical analysis

Data were presented as mean ± S.D. In comparisons of two treatment conditions, two-tailed Student’s *t*-test was used for normally distributed data and Mann–Whitney nonparametric test for skewed data that deviate from normality. In comparisons of three or more treatment conditions, one-way analysis of variance with Bonferroni post hoc test was used for normally distributed data and Kruskal–Wallis nonparametric test for skewed data. Differences with *P* < 0.05 were considered statistically significant.

## Supplementary information

Supplemental Figures
