# Supplementary material for: Identification of a novel class of RIP1/RIP3 dual inhibitors that impede cell death and inflammation in mouse abdominal aortic aneurysm models
Source: Cell Death Dis. 2019 Mar 6;10(3):226. doi: 10.1038/s41419-019-1468-6 (PMC6403222; doi:10.1038/s41419-019-1468-6)
Supplement: Supplementary file 1 — Supplemental Figures [file 41419_2019_1468_MOESM1_ESM.pdf]

Supplemental Figure 1. GSK’074 blocks necroptosis signaling pathway in L929 cells.

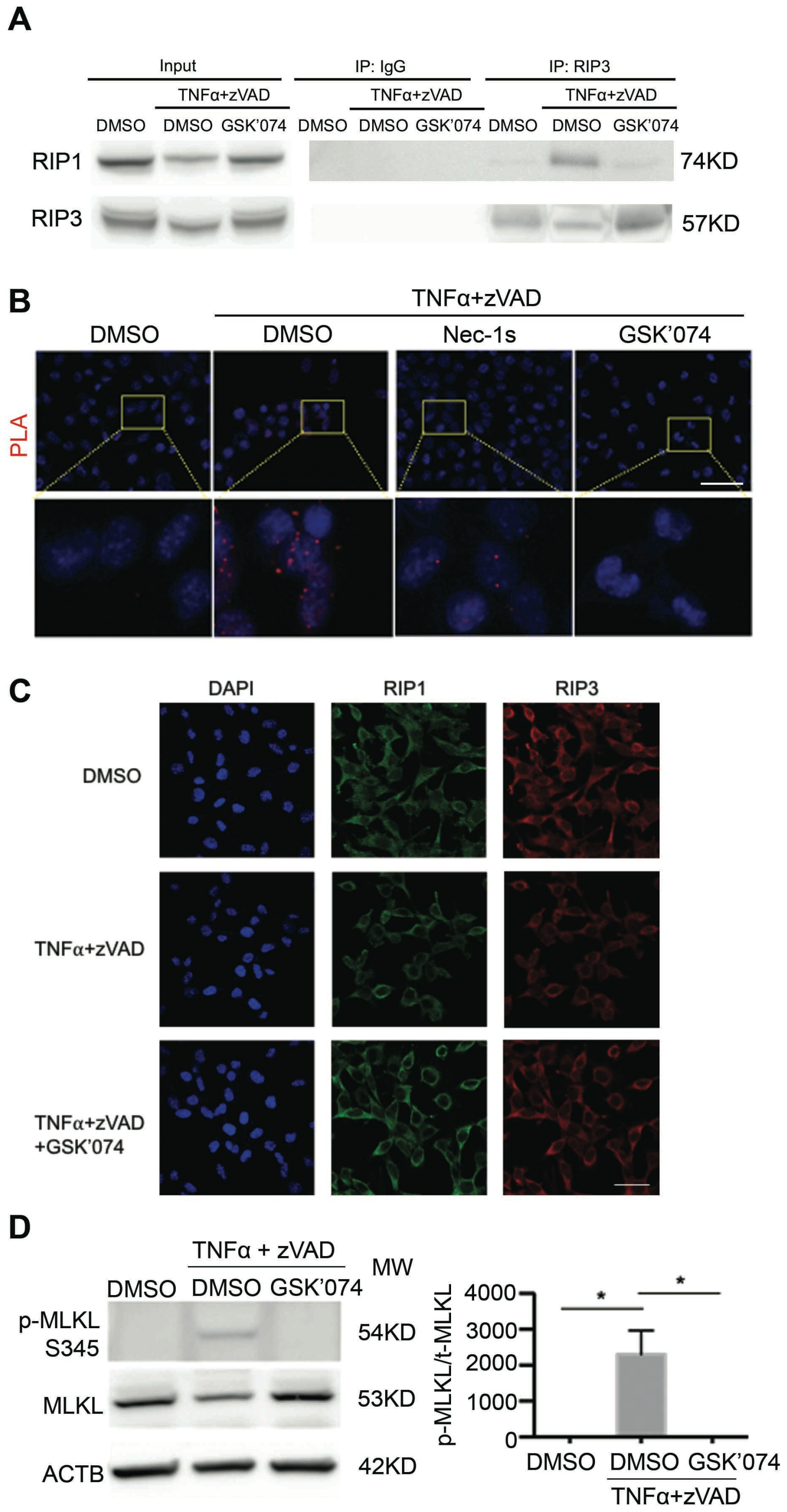

L929 cells were treated with 20ng/ml TNFα plus 40μM zVAD for 3 hours in the presence or absence of GSK’074. RIP1 and RIP3 complex formation were detected by co-immunoprecipitation with an anti-RIP3 antibody followed by immunoblot analysis with the indicated antibodies (A) or by *in situ* PLA assay (B). Cells were fixed and stained with anti-RIP1 and anti-RIP3 antibodies. Representative pictures were shown (C). Scale bar, 50μm. MLKL serine345 phosphorylation (p-MLKL) in whole-cell lysates was evaluated by immunoblot analysis and normalized to total MLKL (t-MLKL) (D).

Supplemental Figure 2. Molecular docking of GSK'843 with RIP3 (DFG-out).

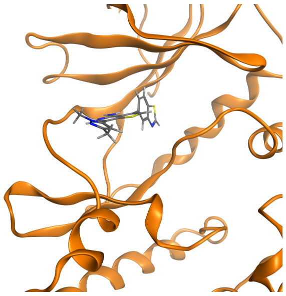

Supplemental Figure 3. GSK'074 blocks toll-like receptor 3 mediated necroptosis.

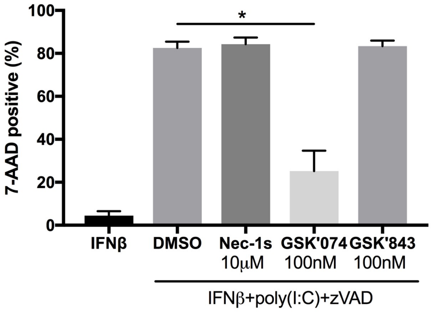

L929 cells were primed with IFNβ (50 units/mL) for 24 hours, then treated with 10 μg/ml poly(I:C) plus 40 μM zVAD and different concentrations of compounds indicated. Cells were stained with 7-AAD and analyzed by flow cytometry. Data represent mean ± S.D. of three independent experiments. *, $p<0.05$.

Supplemental Figure 4. The protective effect of GSK'074 is not due to inhibition of PERK.

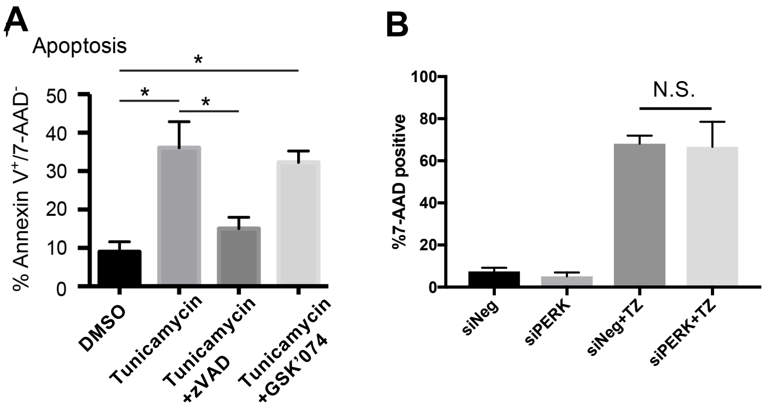

(A) MOVAS were treated with 8 μg/ml tunicamycin plus 40 μM zVAD and 10 μM GSK'074 for 16 hours. Cells were stained with PE Annexin V and 7-AAD and analyzed by flow cytometry. Apoptotic cells were identified as PE Annexin V^+/7-AAD$^-$.(B) L929 cells were pretreated with siPERK or scramble siRNA for 48 hours, then chanllenged with 20 ng/ml TNF α plus 40 μM zVAD and compounds indicated for 4 hours. Cells were then stained with 7-AAD and analyzed by flow cytometry.

Supplemental Figure 5. mRNA level of potential off-target genes in mouse primary SMCs.

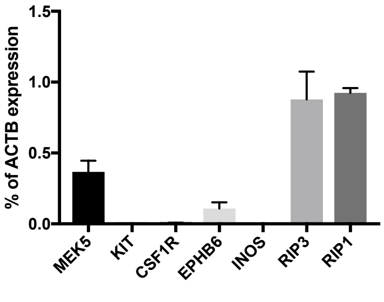

Total mRNA were extracted from primary mouse smooth muscle cells using Trizol, mRNA level of gene indicated were detected by Real-time PCR. Data were normalized to expression of β-Actin (ACTB) and presented as mean value ± S.D. RIP3 and RIP1 were used as comparisons, INOS was used as a negative control which doesn't express in smooth muscle cells. MEK5: mitogen-activated protein kinase kinase 5, KIT:KIT proto-oncogene receptor tyrosine kinase, CSF1R: colony stimulating factor 1 receptor, EPH receptor B6, iNOS: inducible nitric oxide synthase.

Supplemental Figure 6. GSK'414 has weaker anti-necroptotic effect than GSK'074.

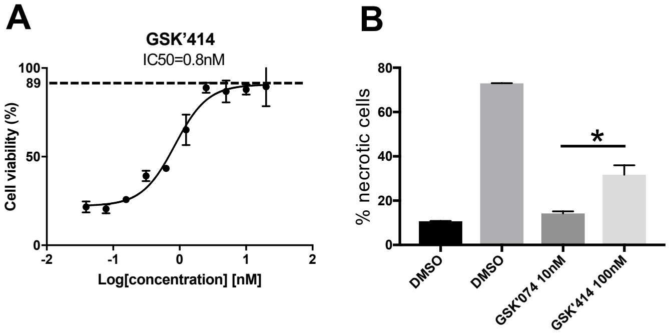

(A) MOVAS were treated with 30 ng/ml TNFα plus 60 μM zVAD and GSK'414 for 6 hours. Cell viability were detected by CellTiter-Glo. Data were normalized to DMSO treated control cells and presented as mean value ± S.D. n=3. (B) MOVAS were treated with 30 ng/ml TNF α plus 60 μM zVAD and indicated compounds for 6 hours. Cells were stained with PE Annexin V and 7-AAD and analyzed by flow cytometry. Necrotic cells were identified as PE Annexin V^+/7-AAD^+.

Supplemental Table 1. Kinases that bind to GSK'074 less than 35% of control (>65% inhibition) detected by KINOME*scan*™

DiscoveRx Gene Symbol	Entrez Gene Symbol	Percent Control	Compound Concentration (nM)
KIT(L576P)	KIT	0	100
RIPK1	RIPK1	0	100
KIT(V559D)	KIT	0.05	100
MEK5	MAP2K5	0.1	100
ABL1(T315I)-nonphosphorylated	ABL1	0.3	100
KIT	KIT	0.35	100
CSF1R	CSF1R	0.5	100
EPHB6	EPHB6	0.5	100
PDGFRB	PDGFRB	1.3	100
ABL1(H396P)-nonphosphorylated	ABL1	2.2	100
ABL1-nonphosphorylated	ABL1	3.3	100
MAP4K5	MAP4K5	5.5	100
ABL1(Q252H)-nonphosphorylated	ABL1	6.1	100
AURKB	AURKB	9.3	100
RIPK2	RIPK2	12	100
MST1	STK4	13	100
PDGFRA	PDGFRA	13	100
ABL1(T315I)-phosphorylated	ABL1	19	100
AURKC	AURKC	19	100
FLT3	FLT3	19	100
ABL1(E255K)-phosphorylated	ABL1	24	100
ABL1-phosphorylated	ABL1	25	100
PFCDPK1(P.falciparum)	CDPK1	27	100
FLT3(K663Q)	FLT3	30	100
HPK1	MAP4K1	30	100
LOK	STK10	30	100
PRKD2	PRKD2	32	100
